# Does the tail show when the nose knows? Artificial intelligence outperforms human experts at predicting detection dogs finding their target through tail kinematics

**DOI:** 10.1098/rsos.250399

**Published:** 2025-08-13

**Authors:** George Martvel, Giulia Pedretti, Teddy Lazebnik, Anna Zamansky, Yuri Ouchi, Tiago Monteiro, Nareed Farhat, Ilan Shimshoni, Dan Grinstein, Yuval Michaeli, Paola Valsecchi, Nathaniel Hall, Sarah Marshall-Pescini

**Affiliations:** ^1^ Department of Information Systems, University of Haifa, Haifa, Israel; ^2^ Department of Medicine and Surgery, University of Parma, Parma, Italy; ^3^ Department of Chemistry, Life Science and Environmental Sustainability, University of Parma, Parma, Italy; ^4^ Department of Mathematics, Ariel University, Ariel, Israel; ^5^ Department of Cancer Biology, University College London, London, UK; ^6^ University of Tsukuba, Tsukuba, Japan; ^7^ University of Veterinary Medicine Vienna, Vienna, Austria; ^8^ University of Aveiro, Aveiro, Portugal; ^9^ Department of Education and Psychology, University of Aveiro, Aveiro, Portugal; ^10^ Department of Evolutionary and Functional Biology, University of Parma, Parma, Emilia-Romagna, Italy; ^11^ Animal and Food Science, Texas Tech University, Lubbock, TX, USA; ^12^ Domestication Lab, Konrad Lorenz Institute of Ethology, University of Veterinary Medicine, Vienna, Austria

**Keywords:** detection dogs, dog olfaction, computer vision, animal behaviour, artificial intelligence, domestic dog, visual communication

## Abstract

Detection dogs are utilized for searching and alerting to various substances due to their olfactory abilities. Dog trainers report being able to ‘predict’ such identification based on subtle behavioural changes, such as tail movement. This study investigated tail kinematic patterns of dogs during a detection task, using computer vision to detect tail movement. Eight dogs searched for a target odour on a search wall, alerting to its presence by standing still. Dogs’ detection accuracy against a distractor odour was 100% with trained concentration, while during threshold assessment, it progressively reached 50%. In the target odour area, dogs exhibited a higher left-sided tail-wagging amplitude. An artificial intelligence (AI) model showed a 77% accuracy score in the classification, and, in line with the dogs’ performance, progressively decreased at lower odour concentrations. Additionally, we compared the performance of an AI classification model to that of 190 detection dog handlers in determining when a dog was in the vicinity of a target odour. The AI model outperformed dog professionals, correctly classifying 66% against 46% of videos. These findings indicate the potential of AI-enhanced techniques to reveal new insights into dogs’ behavioural repertoire during odour discrimination.

## 1. Introduction

The domestic dog (*Canis familiaris*) possesses an extraordinary sense of smell, enabling it to detect and discriminate a vast range of odour compounds, even those in concentrations imperceptible to humans [1]. This remarkable olfactory acuity is supported by a highly developed olfactory cortex [2], dense populations of olfactory receptors [3] and specialized nasal anatomy, including turbinate bones that optimize airflow to odour-detecting areas [4]. Additionally, dogs’ rapid sniffing frequencies (4–7 Hz) enhance odorant detection by optimizing airflow dynamics and entraining odour molecules for efficient delivery to olfactory receptors, thus improving odour discrimination [4].

Dogs’ olfactory acuity and their ability to learn through operant conditioning make them invaluable in detecting various substances, including narcotics and human remains [5]. In training, detection dogs acquire a positive reinforcement history associated with the specific target odour and are taught to signal the odours through passive (e.g. sitting and lying down) or active (e.g. scratching) behaviours. Their performance is commonly evaluated using principles of signal detection theory [6], with outcomes classified as true positives, true negatives, false positives or false negatives. Research suggests that dogs’ sniffing behaviour varies according to these outcomes, with shorter sniff durations for true negatives and longer durations for false positives and other errors [7]. This evidence may suggest that the trained alert response may not be the only element the handler should focus on when interpreting dogs’ performance. In fact, in operational settings (i.e. fieldwork), errors can be due to the dog’s failure to respond correctly to the presence or absence of the target odour and to handler errors in the interpretation of dogs’ behaviour. Dogs’ performance while searching can be hindered if the handler does not recognize the change in behaviour linked with odour perception and, for example, redirects the dog’s attention elsewhere [8,9].

An important aspect of canine behaviour during scent detection is tail wagging. Although the exact function of tail wagging is not fully understood [10], evidence suggests it may reflect dogs’ emotional states, with lateralization to the ‘right’ when dogs are exposed to positive valence stimuli and to the ‘left’ when exposed to negative valence stimuli [11]. Furthermore, a recent study showed that individual dogs exhibit a specific tail asset and moving pattern during an interaction with a stranger human and that this pattern changes after increased exposure (right tail wagging when familiarity increases), suggesting tail lateralization may be an indicator of positive anticipation [12]. Tail wagging occurs not just towards social entities (conspecifics and humans) but also during interaction with the environment [13–15]. In general, tail movement towards non-social entities is considered as an expression of dogs’ positive evaluation of their environment [16]. Considering the above, tail-wagging patterns may be particularly relevant in odour detection scenarios, where, through positive reinforcement training, the target odour acquires a positive valence for the dog. Practical knowledge from trainers and dog handlers suggests that tail wagging (and especially an increase in speed of tail wagging) can be predictive of odour perception during searches. However, this hypothesis has never been tested.

Manual effort is needed to track and analyse dog tail movements from video and automated tools such as artificial intelligence (AI) and computer vision (CV) may help reduce human bias and the time needed for data processing. The field of AI in animal behaviour has historically been behind that of human behaviour, but this is starting to change. Advancements in Computer Vision and Deep Learning have led to the development of general platforms for tracking animal motion and recognizing postures. Examples are DeepLabCut [17], EZtrack [18], DeepPoseKit [19] and others (for a comprehensive overview, see [20]). More recent work has also started investigating the possibility of non-invasive recognition of animal’s affective states [21], providing a powerful tool to further our understanding of nonverbal species. Only a few studies have addressed the recognition of emotional states in facial expressions and body language in dogs and applied automation to analyse dogs’ tail movement and the majority focused on dog-human interactions [10, 12,22–25]. This is ly because of the dogs’ morphological complexity [26], which poses methodological challenges during the data collection phases in order to acquire the high-quality datasets necessary for AI-enhanced analyses. To our knowledge, odour detection scenarios have not yet been explored in this context.

The current study aimed to examine the tail-wagging behaviour of dogs during an odour detection task, with the goal of analysing how kinematic features of tail movement may relate to successful odour detection (i.e. tail movements exhibited prior to the correct trained response being displayed). The set-up was designed to include a number of elements of a real-life detection scenario (and more specifically the presence of distractor odours and different odour concentrations) but within a lab setting, allowing us to collect high-quality video data from a position above the dog, thereby allowing for AI-enhanced analyses. Thus, dogs were tested on a scent wall with 36 holes, divided into three distinct zones: one containing the target odour, another with a distractor odour and a third with no odour. Two testing conditions were conducted: test 1 involved detection at a trained concentration of the target odour, while test 2 gradually reduced the odour concentration (a threshold test) with the prediction that the dogs’ tail movement would reflect the greater difficulty encountered when trying to detect odour at smaller concentrations.

Using top-view videos of these tests, we employed markerless tracking to extract six key body landmarks from each frame, generating precise spatiotemporal data on the dogs’ movements. This enabled us to precisely capture and analyse specific tail kinematic features, including angle, amplitude and velocity. Furthermore, from the spatiotemporal data, we developed a deep-learning model to classify when a dog was in a target odour area based on tail trajectory patterns. The model’s accuracy was tested across both test conditions, allowing us to assess its performance as detection became progressively more challenging for the dogs at decreasing odour concentrations. To further evaluate its reliability, the model’s accuracy was compared with the assessment of 190 experienced detection dogs professionals. This comprehensive analysis represents a first in-depth look at tail movement patterns in canine scent detection, utilizing markerless tracking for accurate kinematic measurement and employing deep learning to evaluate the potential of AI in predicting odour detection success based on tail movement, thereby paving the way for future studies.

## 2. Material and methods

**Figure 1.**
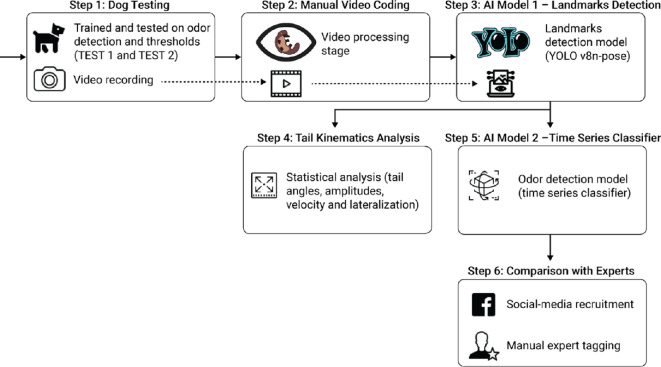
A schematic view of the study’s design. Step 1 (dog testing): dogs were previously trained and then tested on odour detection thresholds (test 1: performed on the trained concentration; test 2: decreasing odour concentrations until reaching detection threshold) with overhead video recording. Step 2 (manual video coding): manual video annotation. Step 3 (AI model 1—landmark detection): training landmarks detection of key dogs’ body from the videos. Step 4 (tail kinematics analysis): statistical analysis of tail kinematic features. Step 5 (AI model 2—time-series classifier): time-series data classification. Step 6 (comparison with experts): detection dog experts’ classifications on selected clips were compared to AI model accuracy.

The study design comprised six steps (figure 1).

In step 1 (dog testing), dogs were trained to detect an odour and then tested first with the trained odour concentration (test 1) and then with progressively lower concentrations (test 2) of the odour. The tests were conducted in the lab, double-blind and with videos being recorded from above.

In step 2 (manual video coding), a manual behavioural annotation was conducted on videos using BORIS software (Behavioral Observation Research Interactive Software) to select and prepare shorter videos that could then be used for the computational analysis, i.e. removing the manifestation of the trained alert response when dogs were on odour.

In step 3 (AI model 1—landmark detection), we applied a CV model (YOLOv8) for landmark detection (three for the dog’s body and three for the tail) to the videos prepared in the previous step.

Based on this output in step 4 (tail kinematics analysis), we statistically compared the tail kinematic features exhibited by dogs when in target and non-target areas in both the test 1 scenario (using the trained odour concentration) and in the increasingly complex test 2 scenario (with decreasing odour concentrations being presented).

In parallel, in step 5 (AI model 2—times series classifier), we used the output from step 3 to develop deep-learning classification models that took the time series as input to predict whether a dog was in a target or in a non-target area in both test 1 and test 2.

Finally, in step 6 (comparison with experts), we asked detection dog professionals to view a smaller subset of videoclips and categorize whether the dogs were searching in a target or non-target area and compared their accuracy to our AI model in correctly classifying the dogs searching behaviour on the same dataset.

### 2.1. Dog testing

Testing was conducted at the Canine Olfaction Research and Education Lab of Texas Tech University. Eight dogs were trained and tested during this study (step 1; figure 1). Four shelter dogs (two females and two males) kept at the Canine Olfaction Lab of Texas Tech University, and four pet dogs (three males and one female) kept as pet dogs that came to the Canine Olfaction Lab for training and testing. The dogs were between 3 and 7 years old, with a mean age of 4.5. Dogs represented different breeds and mixed breeds (for more information, see electronic supplementary material, table S1).

**Figure 2.**
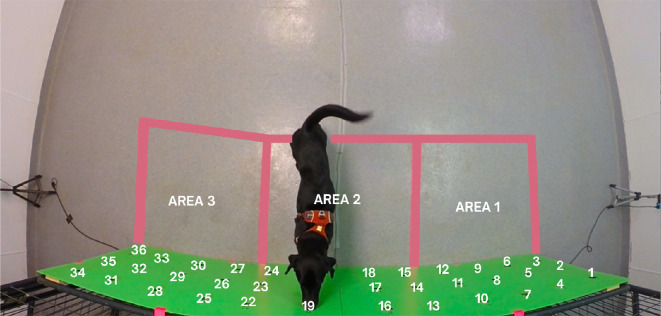
Image displaying the apparatus, testing room and three searching areas.

An indoor room was used for training (approx. 1 month duration) and testing (approx. 2 month duration), as shown in figure 2. Dogs underwent one to two sessions of 10 trial each training or testing day. A hard-wooden wall 1 × 2 m wide and 4 cm thick constituted the base for the scent wall for training and testing of the dogs. Thirty-six holes were present in the wall. The holes functioned as support for 36 aluminium containers used as support for the odours. The area near the search wall was split into three areas to score the time dogs spent searching each area during trials. For further analysis, the *target area* was defined as the area where the target odour was placed in, the *distractor area* was defined as the area where the distractor odour was placed in, while the *no odour area* was defined as the area where no target or distractor odour was present. *Target area*, *no odour area* and *distractor area* changed each trial.

The dogs’ searching and alerting responses were trained using an operant conditioning process (primary reinforcer: food, secondary reinforcer: clicker), through which dogs progressively learned to stand and stare at a container with food for 4 s. The exact same process was successfully performed with the odour container with the target odour: Universal Detection Calibrant (UDC, 1-Broomoctane, a synthetic odour not found in the natural environment, safe for daily use and not toxic for the dogs—see [27]) (TARGET ODOUR = UDC, 10^−2^ dilution factor (volume/volume), diluent = mineral oil and placed (2 ml) in cotton balls). For the training protocol, see the electronic supplementary material, table S1.

Different distractor odours were included in the training process to teach the dog to discriminate between them and the target odour: clean cotton balls (support for each odour), cotton balls + mineral oil, cotton balls + almond oil (10^−2^ dilution factor, diluent = mineral oil). During the training phase, the target and the distractor were placed randomly in one of the 36 holes, respectively. During the test, the target or the distractors were only put in one of the holes of the two middle lines of each area. The handler of the dogs was placed 4 m behind the dog and the wall and was always blind to the location of the target odour. When the dog alerted to a specific hole, the handler called out the number of the hole and a research assistant (placed behind the wall and with their back to the handler) could say whether the alert was correct or not. If the dog performed a correct alert, the handler rewarded the dog with either clicker and food or a toy.

Following training, dogs underwent two tests:

— *Test 1 (detection test)*. Each dog completed 10 sessions, with 10 trials per session. The test environment included target area, distractor odour area and no odour area. The target odour (UDC) and distractor odour (coconut extract) concentration was fixed at a dilution factor 10^−2^, and there was one odour per respective area.
— *Test 2 (threshold test)*. This test environment included a target area and two distractor odour (mineral oil diluent) areas. We performed one session for each concentration of the target odour, lowering it by one log unit in each session until the dog’s performance accuracy was at or lower than 50%. Each session encompassed 10 trials. In this phase, motivational trials with the concentration of the target that dogs could successfully detect were introduced if the dog made a mistake (missed a target odour or performed a false alert on a distractor).

All trials were recorded with a GoPro Hero3+ placed on the ceiling, thereby allowing for a top-down view of the dog in the room (figure 2). The raw footage encompasses 16.6 h of video in 720 p and 60 fps.

#### 2.1.1. Manual video coding

For further AI analyses, video preparation was required in order to select video clips of dogs searching in the target and non-target areas, removing the frames in which dogs performed the trained alert response (step 2, figure 1). Therefore, an independent annotator manually coded each video for four exclusive behaviours based on ethogram definitions (table 1) using the BORIS platform [28]. Start and end times were coded for each behaviour.

**Table 1.** Behavioural ethogram used for manual video coding for video clip preparation.

behaviour	description
area 1	the dog has at least one of the front paws or a part of the head inside area 1 (the first area on the left side of the wall, from the dog’s perspective, in the video)
area 2	the dog has at least one of the front paws or a part of the head inside area 2 (the area in the middle of the wall in the video)
area 3	the dog has at least one of the front paws or the head inside area 3 (area on the right side of the wall, from the dog’s perspective, in the video)
alert	the dog kept its nose touching or close to the hole for at least 4 s; the dog could completely freeze in front of the hole or could move parts of its body, as long as its nose was kept in position pointing towards the hole.

In total, 1110 videos were coded and then processed: each video was split into shorter videos based on when the dog was searching in a specific area and removing videoclips starting 1 s prior to the dog exhibiting the alert behaviour and including all the time the dog continued to exhibit the alert behaviour. This processing resulted in 5055 pre-processed videos (3.34 ± 3.45 s, 3375 from test 1 and 1680 from test 2), each with the dog searching only in a single area. The videos were then classified as ‘target’ or ‘non-target area’ (the latter including both the empty area and area containing the distractor odour) based on the position (area 1, area 2 and area 3; figure 2) of the target odour in each trial.

### 2.2. Artificial intelligence model—landmark detection

A CV model was trained to detect six landmarks on the dog’s body in order to gather coordinates of the corresponding body parts in each frame of the processed videos (figure 1, step 3). The coordinates of the tail (landmarks 4−6, see below) were then used to (i) statistically analyse differences in kinematics features (i.e. angle, amplitude and velocity) of the tail when the dog was in the target and non-target area (figure 1, step 4) and (ii) to extract spatiotemporal data to train the time-series classifier (figure 1, step 5) to predict when the dog was in a target or non-target area.

**Figure 3.**
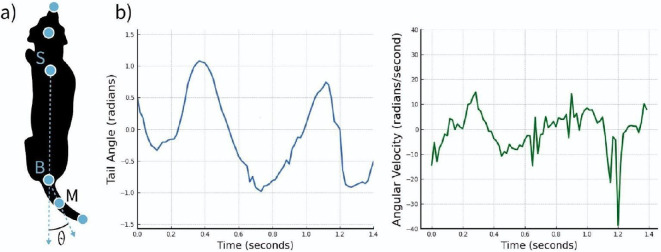
(a) Schematic representation of a dog with a coordinate system and tail-wagging angle *θ*. (b) An example of the dynamics of an angle (left—negative angle) and angular velocity (right—positive angle) of the tail middle landmark during a random trial.

Starting with landmark detection, we retrieved 5488 random images from all trial videos, excluding those on which the dog was not visible. This resulted in 3756 images annotated with six landmarks using the Labelbox platform [29]. The annotation landmark scheme included the following (figure 3a):

— Landmark on the nose tip labelled *nose*.
— Landmark on the head between the two ears, labelled *front head*.
— Landmark on the body between the shoulders, labelled *shoulders*.
— Landmark where the body meets the tail, labelled *tail base*.
— Landmark in the middle of the tail, labelled *tail middle*.
— Landmark on the tip of the tail, labelled *tail tip*.

The images were split into the train/test sets with a 90 : 10 ratio. For landmark detection, YOLOv8n-pose was used (as it was the latest version of the YOLO model at the time the analysis was conducted) with the default parameter values.

We used two metrics on the test set to evaluate the detection performance: root mean square deviation (RMSE) and percentage of correct predictions when threshold is set at 10% (PCK@0.1). RMSE measures the Euclidean distances between the predicted landmark positions and their corresponding ground truth coordinates averaged across all landmarks


$$RMSE=\;\sqrt{\frac{1}{n}\sum_{i=1}^{n}{\left(y_{i}-{\hat{y}}_{i}\right)}^{2}},$$

where $y_{i}$ denotes the predicted value for the *i*-th landmark, ${\hat{y}}_{i}$ denotes the true value for the *i*-th landmark and *n* represents the total number of landmarks assessed. PCK@0.1 measures the percentage of predicted landmarks that fall within a threshold distance from their true positions, normalized by a specific anatomical measure to account for varying scales across images or subjects


$$PCK@0.1\;=\;\frac{100}{N}\sum_{i=0}^{N}I(d(p_{i}\;,{\hat{p}}_{i})\leq 0.1D),$$

where $p_{i}$ and ${\hat{p}}_{i}$ are the predicted and true positions of the *i*-th landmark, respectively, $d\left(p_{i},{\hat{p}}_{i}\right)$ is the Euclidean distance between them, D is the normalization distance (shoulders-tail base distance), N is the total number of landmarks and $I\left(\cdot \right)$ is an indicator function that yields 1 if the condition within the brackets is satisfied, and 0 otherwise. The threshold of 0.1 typically means that a landmark is correctly predicted if it is within 10% of the normalization distance D from its true location.

After the final training iteration, we evaluated the landmark detection model on the test set, obtaining RMSE and PCK@0.1 of 11.13 and 84.04, respectively. Then, we detected landmarks on the pre-processed videos using our model. For each frame, our model predictions are represented as follows: timestamp; landmarks (*x*, *y*) for (nose, front head, shoulders, base of the tail, middle of the tail, tip of the tail); and model’s confidence. It is important to note that despite the fact that we trained a model to detect six body landmarks, only four of them were used for subsequent analyses. We initially intended to use them for PCK normalization, but found them too unstable for that purpose, instead switching to *shoulders* landmark, as described above. Thus, neither *nose* nor *front head* landmarks were used in tail kinematic analysis and in the time-series classification.

### 2.3. Tail kinematics analysis

We used the coordinates of the *shoulders* landmark and three landmarks of the tail (*tail base, tail middle* and *tail tip*) to calculate tail kinematic features (figure 1, step 4). To do so, we first normalized landmarks and transferred their coordinates to a coordinate system in which the centre was the tail base landmark (B) and aligned the *y*-axis with the shoulders-tail base axis (SB), as shown in figure 3a.

We calculated the angle between the SB axis and a vector directed through the middle of the tail (BM) to quantify tail movement. The differences in tail angle values between consecutive frames were used to compute the angular velocity of the tail middle landmark. Figure 3b shows an example of the tail angle *θ* and angular velocity dynamics during a random trial.

We focused on the tail middle rather than the tip for stability reasons, as the tip tends to exhibit excessive mobility that does not always correspond with the overall direction of tail wagging, particularly in breeds with curly tails. We summarized the tail’s spatial distribution using the angle data, identifying regions where the tail was predominantly positioned. For this, we divided the semicircle around the dog into sectors and computed a histogram of the cumulative tail middle landmark positions over time.

The kinematic features were defined based on the tail angle *θ*, angular velocity and polar distribution. They include (1) mean angle, (2) amplitude positive, (3) amplitude negative, (4) mean absolute velocity, (5) sum positive, and (6) sum negative:

(1) *Mean angle* (*mean angle*). The average tail angle over the duration of the search in each video.
(2) *Maximum positive amplitude* (*amp positive*). The highest amplitude of tail movement to the right (relative to the dog’s perspective).
(3) *Maximum negative amplitude* (*amp negative*). The highest amplitude of tail movement to the left.
(4) *Mean absolute velocity* (*mean abs velocity*). The average speed of tail movement, calculated as the mean of the absolute angular velocity.
(5) *Sum of positive tail angles* (*sum positive*). The total number of frames in which the tail angle was positive, divided by the total number of frames of the video (indicating movement or positioning to the right).
(6) *Sum of negative tail angles* (*sum negative*). The total number of frames in which the tail angle was negative, divided by the total number of frames of the video (indicating movement or positioning to the left).

Features related to positive and negative angles provide insights into lateralization, reflecting the directional bias of the tail in different areas. Specifically, *sum positive* and *sum negative* quantify the proportion of time (measured in video frames) that the tail spent positioned to the right or left, respectively, offering a measure of lateralization. Meanwhile, *maximum amplitude* captures the extent of tail movement to the far right or left, providing a metric of the range of tail motion during the search task.

To investigate whether dogs exhibited specific tail movement patterns in response to the presence of a target odour, we conducted generalized linear mixed models with tail kinematics features as response variables and the factor ‘Area’ (categorical: *no odour area*, *target area* and *distractor area*) as the primary predictor. The kinematic parameters analysed included mean angle, mean absolute velocity, maximum positive amplitude, maximum negative amplitude and the sum of frames with the tail on the right. *Session number* and *trial number* were included as control fixed effects, while *Dog ID* was entered as a random effect to account for repeated measures within individuals.

A Gaussian error distribution was applied to models with continuous variables (mean angle, mean velocity and maximum amplitude) as response variables, while a beta distribution was used for proportion of *sum right* and *sum left* (proportion of the frames in which the tail was on the right or on the left divided by the total number of frames). Analyses were conducted for both experimental tests (test 1 and test 2 separately).

To further investigate whether kinematic parameters varied within individual dogs while searching in different areas, we performed linear mixed models for each parameter for each dog during test 1. Session and trial were retained as control fixed effects in these models.

We assessed collinearity among predictors using the variance inflation factor function from the *car* package (v. 3.0-0), excluding random effects. Model stability was evaluated by sequentially excluding levels of the random effect, confirming stable estimated coefficients and s.d. (see electronic supplementary material for details). Confidence intervals were generated through parametric bootstrapping (function ‘boot.glmmTMB’), allowing for control of individual variability and a robust assessment of experimental conditions on tail kinematics.

### 2.4. AI model 2—time-series classifier

This analysis (step 5, figure 1) consisted of training deep-learning models to classify whether the dogs were searching in a target or non-target area based on the movement of the tail, thus picking up on cues of the dogs’ tail movements when detecting the target odour. To do so, we considered only the spatiotemporal data (time series: timestamp, *x* and *y* coordinates, model’s confidence) from the three tail landmarks (*tail base, tail mid* and *tail tip*). For the training of the classifiers, only videos of the target area from trials in which dogs successfully alerted to the target odour were included.

Since tests 1 and 2 had different smell configurations, we did not mix the samples from these two tests for training. Instead we opted for training two different classifiers for test 1 and test 2. Importantly, in both cases, we applied a leave-one-out cross-validation technique. In each iteration of training, the model was trained on all but one dog’s samples, which were reserved for testing. This process was repeated for all dogs, allowing the model to encounter an unseen dog in every testing phase. The final performance was calculated as the average accuracy across all iterations. This method is adopted to ensure that the model’s performance is not biased towards dog-specific patterns but rather generalized to broader behaviour patterns.

#### 2.4.1. Classifier for test 1

For test 1, we divided the data into five pairwise distinct sets following a *k*-fold cross-validation method [30] such that 80% of clips were used as the training set, while the remaining (20%) were used as the test set. We then trained a recurrent neural network (RNN) model with five layers. The input layer accepts seven values corresponding to the normalized location of the three points on the dog’s tail and the model’s confidence regarding these points. Next, a gated recurrent unit (GRU) layer with 32 dimensions is used to capture the temporal dynamics of the landmarks, followed by another GRU layer with 16 dimensions responsible for extracting higher-level temporal information. Next, we combined three fully connected (FC) layers with 16, 8 and 4 dimensions (all with a rectified linear unit activation), followed by a dropout with a rate of *p =* 0.1*.* These layers are responsible for finding a high-dimensional and nonlinear relationship between the input data’s change over time and the target variable. The dropout layers are included to ensure a better generalization due to the relatively small dimension of the data. Finally, the output layer is consistent with an FC layer with one dimension due to the binary nature of the classification task, with a sigmoid activation function. For the training process, we used binary cross-entropy for the loss function and adam, an algorithm for first-order gradient-based optimization of stochastic objective functions [31], as the optimizer. Importantly, to ensure an imbalance in the dataset does not infect the training process, we weighed the class’s importance in the loss function to be their portion of the entire training set (i.e. 0.001).

#### 2.4.2. Classifier for test 2

For test 2, we used the videos of the dilution factor 10^−3^ testing sessions to train the model and the videos from sessions with lower odour concentration (dilution factor 10^−4^, 10^−5^ and 10^−6^) as a testing set. In test 2, the RNN model used for test 1 did not converge, which can be explained by the limited amount of data. We therefore adopted the Tree-based Pipeline Optimization Tool (TPOT) [32] for this case, an automatic machine learning framework using genetic algorithms. We set the population size to 50 and the number of generations to 40, practically searching over 2000 machine learning pipelines, following common practices [33]. Due to the limited amount of training data, we did not use cross-validation.

In each iteration, we evaluated the models on the test set using accuracy (how often a model correctly predicts the right answer out of all its predictions) and F1 score (a harmonic mean of precision—how many of the positive predictions are actually correct—and recall—how many of the actual positive cases the model successfully found, see [34]). For test 1, the final accuracy and F1 score are these values’ mean (average) across all folds, while in test 2, there were no folds due to the small size of data.

#### 2.5. Comparison of model accuracy and human experts

To evaluate and compare the performance of the automated model with that of expert dog handlers in recognizing when dogs were in target odour areas (figure 1, step 6), we conducted an online survey in order to obtain the data from dog handlers. Experienced detection dog handlers were invited to classify the same video dataset presented to the AI. Experts could re-watch the video as many times they liked before the classification, and videos were presented in a randomized order.

We selected four random videos per dog from the dataset of the shortened video clips processed in step 2 (all video clips were from test 1). Two of these were chosen from those in which the dog had then successfully alerted to the target odour, and the other two represented the dogs searching in non-target areas (for a total of 32 video clips, from them 16 in target, 8 in control odour and 8 in no odour areas). Video durations ranged from 1.2 to 19.3 s, with an average length of 5.3 ± 4.2 s.

To ensure a fair comparison between the model and the handlers, the classification model was trained on the entire dataset, excluding the selected subset of videos used in the expert survey. This approach ensured that both the model and human participants classified the same video that had also not been previously seen by the AI model, thereby minimizing a potential bias.

The survey was built using the Qualtrics web platform (https://www.qualtrics.com), and participants were instructed to label each video as either ‘*Trained target odor IS present in this region*’ or ‘*Trained target odor is NOT present in this region*’. They were also given the option to skip any video if unsure.

In addition to assessing classification accuracy, the survey collected demographic and professional information, including the participant’s age, the nature of their detection dog experience, years of paid detection dog handling experience and continent of residence (see electronic supplementary material, ‘Survey Details’).

To evaluate the performance of both the AI model and human experts, we compared areas under the receiver operating characteristic curve (AUCs) with the DeLong test [35]. This approach is commonly used to compare machine and human performance in classification tasks in the clinical domain (e.g. [36]). The AUC represents a standard index to evaluate classification performance, varying from 0 to 100.

## 3. Results

### 3.1. Dogs’ performance

In test 1, five dogs (Bubba, Buster, Charlie, Dasty and Luna) consistently performed with 100% accuracy, exhibiting no misses or false alerts across all trials. Of the remaining dogs, Sam achieved an average accuracy of 96%, Riot 99% and Dot 98%.

In test 2, with decreasing odour concentrations, we found that seven dogs reached the 50% detection threshold at 10^−6^ dilution factor, while one dog (Sam) reached threshold at 10^−4^ dilution factor (see electronic supplementary material, table S2 for dogs’ performance results).

### 3.2. Statistical analysis of kinematic features

**Figure 4.**
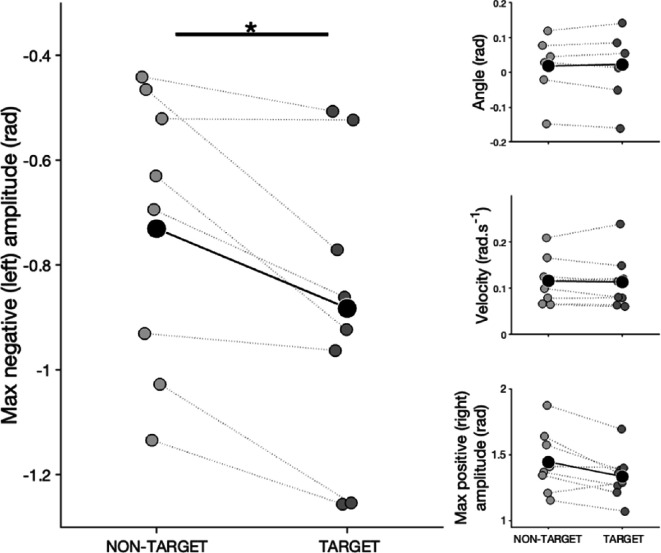
Kinematics features profiles during test 1. Across panels, large markers depict group means and smaller markers individuals for non-target (left) and target areas (right). *p < 0.05.

In test 1, the only kinematic parameter significantly influenced by the area factor was maximum negative amplitude (figure 4; comparison with full-null model: *χ*² = 7.323, *p* = 0.026). Specifically, dogs exhibited higher negative (left-sided) amplitude in the target area compared to the no odour area (Es = 0.088 ± 0.035, *z* = 2.498) but no difference was found between the target area and the distractor area. Being in the target area did not influence other kinematic parameters: tail angle (comparison with full-null model: *χ*² = 1.324, *p* = 0.516), tail velocity (comparison with full-null model: *χ*² = 2.061, *p* = 0.357), max amplitude positive (comparison with full-null model: *χ*² = 5.071, *p* = 0.079), sum right (comparison with full-null model: *χ*² = 1.671, *p* = 0.434), sum left (comparison with full-null model: *χ*² = 1.671, *p* = 0.434; see electronic supplementary material for models outputs).

Individual-level analyses for test 1 revealed that only a subset of dogs (three out of the eight dogs tested) exhibited kinematic tail patterns significantly influenced by the area:

— *Bubba*. Maximum negative (left-sided) amplitude was higher in the target area compared to the distractor area (Es = 0.166 ± 0.070, *t* = 2.369, *p* = 0.049), no differences between the target area and the no odour area.
— *Sam*. Tail velocity was lower in the target area compared to both the distractor area (Es = −0.011 ± 0.004, *t* = −2.894, *p* = 0.011) and the no odour area (Es = −0.008 ± 0.004, *t* = −2.162, *p* = 0.079).
— *Riot*. Maximum positive (right-sided) amplitude was higher in the target area compared to the no odour area (Es = 0.367 ± 0.110, *t* = 3.311, *p* = 0.030), but no significant difference was observed between the target and distractor areas. Sum of frames with the tail on the right (sum right) was higher in the target area compared to the no odour area (Es = 0.487 ± 0.156, *t* = 3.311, *p* = 0.005) and marginally higher in the target area compared to the distractor area (Es = 0.341 ± 0.150, *t* = 2.267, *p* = 0.06).

In test 2, maximum positive (right-sided) amplitude was significantly affected by the area where the dog was searching (comparison with full-null model: *χ*² = 13.502, *p* < 0.001). Dogs displayed lower positive amplitude (right-sided wagging) in the target area compared to the distractor area (Es = −0.172 ± 0.047, *t* = −3.691). Because of the reduced data points, we did not carry out the individual-level analyses for test 2.

Overall, results indicate that while group-level differences in tail kinematics were modest, some individual dogs displayed distinct tail movement patterns depending on the search area, suggesting variability in behavioural responses to odour cues.

### 3.3. Performance of the classifier model

**Figure 5.**
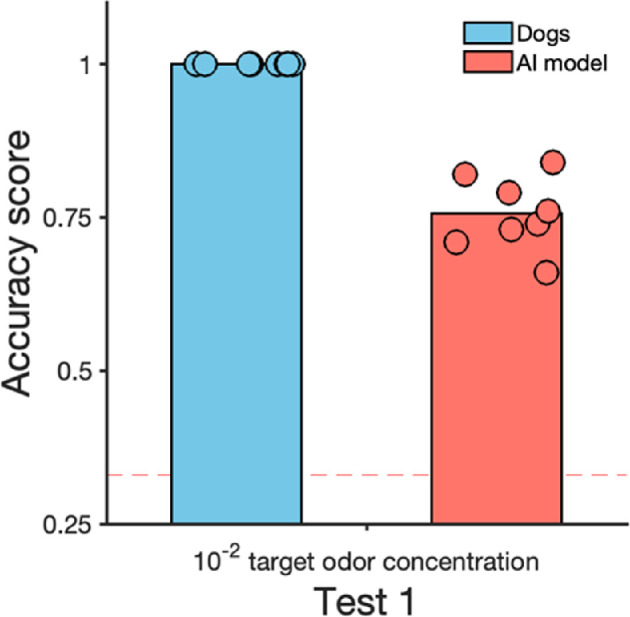
Dogs’ accuracy in signalling the target odour and the AI model’s accuracy in predicting whether dogs were in the target odour area or not, in test 1. Three area types were present: target, distractor and no odour. Chance level is indicated with the dotted line at 33%.

In test 1, our model achieved an accuracy of 0.77 (and F1 score of 0.74) in classifying whether dogs were in the target area or not (in test 1, dogs had three potential areas in which they could be; therefore, chance level could be considering 0.33—see figure 5). Metrics per individual dog ranged between 0.64 (with Dot being the most difficult dog for the classification task) and 0.81 (with Luna being the easiest one).

**Figure 6.**
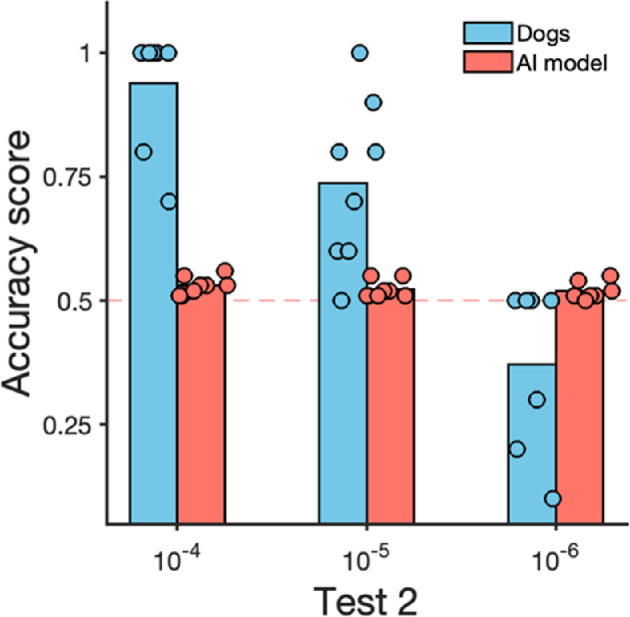
Dogs’ accuracy in signalling the target odour, and the AI model’s accuracy in classifying whether dogs were in the target versus distractor odour areas in test 2 (with decreasing concentrations). Odour concentration 10^−3^ dilution factor was used for training and is not shown here. Three areas were present, but one contained the target odour and the other two contained the distractor odour, thus chance level is indicated with the dotted line at 50%.

For test 2, the TPOT automatic machine learning framework found that an ensemble of XGboost model (depth 9) with support vector machine model (radial basis function kernel) with weighted majority vote is the best machine learning pipeline for the task. Based on this model, when the odour concentration progressively decreased, the AI model’s performance dropped below chance levels (from 0.54 accuracy at 10^−4^ 0.01%, 0.52 accuracy at 10^−5^ and 10^−6^ dilution factor; figure 6).

## 4. Comparison to human expert baseline

One hundred and ninety dog handler experts, ranging in age from 18 to 84, participated in the survey (see electronic supplementary material for detailed information on survey participants and their responses). Considering only the videos the participants chose to classify, the average accuracy in classifying whether a dog was in the target area across all participants was 46%, with the AUC = 0.41. When comparing participants with no paid experience as detection dog handlers (*n* = 90) to those with professional experience (*n* = 83), there was little difference in performance, with accuracies of 47 and 45%, respectively.

Interestingly, approximately 10% of respondents indicated in the commentaries that they found it difficult to assess dog behaviour from short video clips and suggested that longer videos might be needed to capture behaviour changes related to odour detection. However, the survey results indicated the opposite; there was a weak negative correlation between video length and classification accuracy (−0.31), suggesting that longer videos did not improve participants’ ability to classify correctly.

The model’s performance, on the same subset of videos used in the expert survey, showed a 66% accuracy, with an AUC of 0.52. Although the model’s performance was lower than the accuracy obtained on the original test set (77%), this is not surprising considering the number of videos was much smaller, a choice made to guarantee the participation of dog professionals in the survey. The DeLong test comparing the AUCs of the model (0.52) and the human experts (0.41) showed that the model was significantly more accurate in identifying the dogs’ presence in the target odour area (*p* < 0.01).

**Figure 7.**
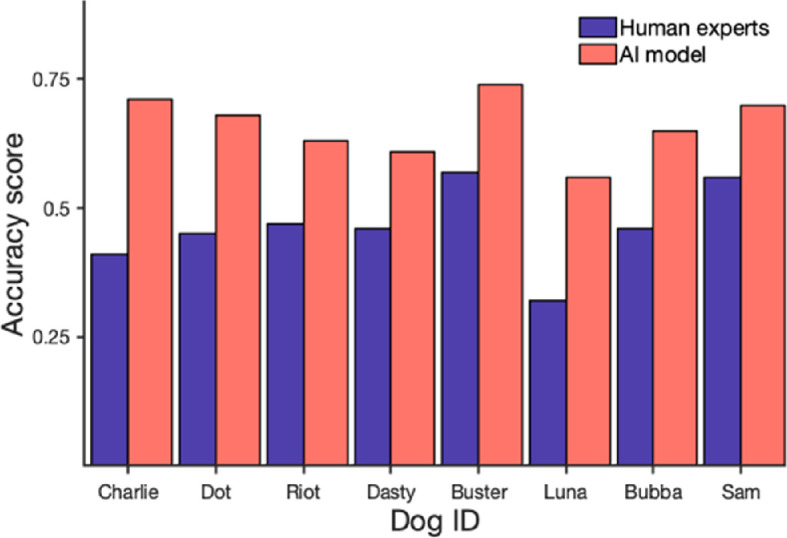
Accuracy in classifying whether dogs were in the target odour area or not by professional experts and the AI model.

Both the human and AI models’ mean accuracy showed considerable variability across dogs tested (figure 7); however, the performance of experts and AI model showed a moderate-to-high positive correlation of 0.72 (*p* < 0.05), suggesting some dogs are easier than others for both.

## 5. Discussion

Our study demonstrates the potential of using AI-assisted behavioural analyses to evaluate dog behaviour during scent detection tasks. The developed deep-learning model successfully identified behavioural differences between target and non-target odour areas based on tail movements, with an overall classification accuracy of 77%. And even on a substantially smaller dataset, the AI model’s performance of 66% accuracy outperformed that of professional detection dog trainers on the same dataset (which was at 46%). Finally, the model showed a predicted decrease in accuracy when analysing dogs’ behaviours when searching at lower concentrations, when indeed the dogs themselves were struggling to identify the odour. Taken together, these results highlight the potential of using an integrated approach, including AI models, to evaluate the behaviour of detection dogs in such tasks.

The kinematic analyses of tail-wagging features, however, revealed only relatively minor differences when dogs were searching target and non-target areas. Negative (left side) wagging amplitude was higher when dogs were in a target compared to a no-odour area, but no differences emerged between target and distractor areas. Furthermore, in test 2, dogs displayed lower positive amplitude (right-side wagging) in the target area compared to the distractor area. These results are not in line with our predictions. Based both on the suggestions by professional detection experts and on the albeit-limited scientific literature on tail movement and lateralization, we had expected differences in both the velocity and lateralization (more right-sided wagging) behaviour, when dogs were in the target odour area, with both tail patterns being considered indicators of positive anticipation [11,37]. Although our results did not confirm our predictions, it is interesting to note that until now studies on the lateralization of tail wagging have presented only social stimuli (owner, human stranger, dog stranger or cat) or no stimuli to dogs, thus it is possible that tail wagging lateralization is related rather to the social than non-social context and functions as a communicative signal [37], in fact seeing conspecific left or right asymmetric tail wagging elicit different emotional responses in dogs [38].

There may be a number of reasons for these limited differences. First, the individual-level analysis revealed that only three (of the total eight) dogs showed differential tail-wagging patterns when searching in the target and non-target odour areas and only one dog, Riot, showed a pattern of behaviour consistent with our predictions (higher velocity and a right-sided wagging when in a target odour area). Thus it seems that tail-wagging patterns are highly individualized, a result that has also been observed in past studies [12].

A second aspect to consider is that the analyses on the tail kinematics included multiple video segments of the dogs during the search in both the target and non-target areas, but only a minority of these will have actually represented dogs both being in the target area and being ‘in-odour’. In other words, dogs may have been in the target area, but not picking up the odour in some of the videos for that area, hence showing ‘no odour’ tail movements. The current set-up did not allow for a more precise distinction between such occurrences. Although this increased the difficulty for the AI model, it was more representative of a real-life scenario. Future studies with more localized target areas (e.g. ‘line-up’ scenarios) may allow for increased precision in distinguishing between tail kinematics when dogs are ‘in or out of odour’ and potentially reveal different results.

Third, the current results are based on a two-dimensional analysis of the tail movement, i.e. velocity and lateralization; however, we were unable to include tail elevation in the analyses because of the limitation of the top-down video angle. This aspect should also be considered in future studies, for example, by using a depth-camera that enables measuring the distance between the camera and objects in its field of view.

A final consideration relates to the potential influence of the trained indication. The decreased tail amplitude shown in test 2 when in the target area may suggest that dogs showed more restrained tail movements in response to detecting a target odour. This effect could be also a consequence of getting ready to exhibit the trained alert response which for these dogs was standing still for 4 s.

Nevertheless, despite results on the kinematics of the tail-wagging showing few specific differences elicited by target and non-target odours, the AI model was largely successful in predicting whether dogs were in the target areas or not, especially in test 1, when dogs were alerting to the odour with very high levels of accuracy. Interestingly, the accuracy of the model varied across individual dogs, with tail-wagging patterns of some dogs (e.g. Luna, Sam) in target and non-target areas being better predicted by the model compared to the tail-wagging patterns of others (e.g. Dot). This variability can be explained by the individual-specific tail movement patterns noted in this and previous studies [12].

Another notable finding is that the model’s performance declined as odour concentration decreased. Two aspects of interest emerge. First, these results suggest that the dogs’ behavioural cues (tail features specifically) become subtler at lower intensities, making it difficult for the model to detect when dogs were searching in the target odour area. Thus, at the threshold of odour concentration, dogs’ behavioural cues, including tail movements, may not be reliable indicators of odour detection.

The second aspect is that including a more ‘real-life scenario’ with increasing complexity levels for the dogs and finding a corresponding decrease in the model’s performance actually increases our confidence in the AI models’ abilities to detect a ‘real’ phenomenon. Thus, although the AI is a ‘black box’, in that it is not possible to know exactly what parameters are being used in making predictions, testing the model in scenarios of differing difficulties can be considered a validator of its honesty. The model’s decreased performance with the increasing task complexity confirms that its success was based on the tail features being exhibited when dogs were highly confident in their responses (i.e. in test 1). Interestingly, however, the three dogs that had shown significant differences in their tail-wagging features in target versus non-target areas based on the kinematic analyses (i.e. Riot, Sam and Bubba) were not the easiest for the model to accurately classify (figure 6), suggesting that the model used parameters beyond the identified kinematic features we identified or used them in a combination which we could not analyse with our current methods.

Similarly, the comparison with detection dog professionals was aimed at further evaluating the AI model’s capacity to correctly identify the dogs’ behaviours. It is important to note that the AI model was put at a disadvantage by substantially reducing the available dataset on which it could be trained. The reduced number of videos was a necessity in order to guarantee voluntary participation by dog professionals, and it was deemed important for the comparison that both dog experts and the AI model performed the evaluation on the same dataset, with neither party having prior experience with the videos.

Results showed that although the AI models’ performance decreased from 77 to 66% accuracy on this smaller dataset, it was still significantly higher than the 46% accuracy rate by the dog professionals. Additionally, several participants expressed difficulty interpreting the dogs behaviour due to the video being too short and from a top-down perspective, which is an unfamiliar angle from which to view dogs for handlers. The weak negative correlation between video length and classification accuracy suggests that increasing video duration alone would not necessarily have improved the ability to detect behavioural cues associated with scent detection in this particular study. However, it remains to be seen if indeed a more familiar side-view of the dogs would have improved the classification accuracy by the dog professionals.

Considering the individual-level variability, it is possible that handler performance would improve with greater exposure to videos of the specific dogs finding and not finding the odour, allowing the humans (as well as the AI model) to obtain a better understanding of how each single dog reacts when finding the target odour. Results highlight the challenges in the subjective interpretation of canine behaviour [39,40]. Interestingly, there was a moderately positive correlation in the classification success of the individual dogs’ performance carried out by the model and the experts, suggesting the same dogs were harder for both.

## 6. Conclusion

Overall, our findings suggest that automated tracking of tail movements, combined with time series and statistical analysis, offers a promising approach to enhancing our understanding of canine scent detection behaviour. This approach could improve detection dog training by providing handlers with additional, objective behavioural cues in real time. However, given the variability among dogs and the challenges in interpreting behavioural responses at low odour concentrations, future research should focus on refining individual-specific models and exploring other aspects of canine body language that may further aid in odour detection tasks.

## Data Availability

The dataset supporting the findings of this study is openly available on Dryad [41]. The dataset includes processed time-series data of detected landmarks, along with associated metadata and source code for data processing and analysis. Original video recordings used for landmark extraction are available upon request to the corresponding authors. Supplementary material is available online [42].
